# Is young children’s helping affected by helpees’ need? Preschoolers, but not infants selectively help needy others

**DOI:** 10.1007/s00426-019-01148-8

**Published:** 2019-02-13

**Authors:** Markus Paulus

**Affiliations:** Ludwig-Maximilians-Universität München, Munich, Germany

## Abstract

Infants and toddlers engage in instrumental helping, that is, help others in achieving an action-based goal. The underlying psychological mechanisms are unclear and hotly debated. The present study examined whether young children’s helping is affected by others’ need. To this end, 1.5- and 3.5-year-old children (*n* = 101) were simultaneously confronted with a needy and a non-needy other in a variety of helping tasks. The results show that the 3.5-year-old, but not the 1.5-year-old children preferentially helped the needy person. This suggests developmental changes in the psychological mechanisms underlying early instrumental helping. The results are explained by a developmental account according to which helping only gradually becomes an other-oriented and need-based behavior in the first years of life.

## Article highlights

- We examined the nature of young children’s instrumental helping.
- 1.5- and 3.5-year-olds were confronted with a needy and non-needy other in several classical helping situations.
- 3.5-, but not 1.5-year-old children helped more the needy than the non-needy other.
- The psychological mechanisms underlying young children’s helping change during the first years of life.

## Introduction

Recent research has experienced increased interest in the psychological basis of social coordination and joint action (e.g., Knutsen & LeBigot, 2018; Müller et al., 2011; Sebanz, Bekkering, & Knoblich, 2006; Yamaguchi, Wall, & Hommel, 2017, 2018). This interest was sparked by theoretical considerations that the ability to act jointly with others is fundamental for human social life (e.g., Bratman, 1992; Gilbert, 2009) and lies at the basis of human cultural evolution (Habermas, 1985; Richerson & Boyd, 2005).

Different forms of social coordination emerge early in development. For example, by 2–3 months infants adapt their posture when their mother approaches to pick them up (Reddy, Markova, & Wallot, 2013). In the course of the first year, infants increasingly engage in routinized play activities with their caregivers (e.g., Fantasia, Fasulo, Costall, & Lopez, 2014; Gustafson, Green, & West, 1979; Ross & Lollis, 1987), such as peekaboo games (Bruner & Sherwood, 1976). By the end of the first year, children engage in a variety of coordinated activities with others. For instances, they coordinate their attention to persons and objects to establish episodes of joint attention (Bakeman & Adamson, 1984; Moore, 2006) and they coordinate their bodily activities when being engaged in self-care tasks with their parents (Hammond, Al-Jbouri, Edwards, & Feltham, 2017). These early coordinated and synchronized activities have been suggested to play a central role in infants’ developing social understanding and language acquisition (Reddy & Uithol, 2016; Ratner & Bruner, 1977).

One form of early social coordination that has recently received considerable attention in the literature concerns so-called instrumental helping. That is, infants have been shown to coordinate their activities with others in such a way that it appears to be helpful in reaching an action-based goal. Expanding on earlier observational work (e.g., Rheingold, 1982), current experimental work provided evidence for such helpful behaviors in a variety of situations (e.g., Barragan & Dweck, 2014; Dunfield & Kuhlmeier, 2013; Kenward & Gredebäck, 2013; Svetlova, Nichols, & Brownell, 2010; Warneken & Tomasello, 2006). For example, infants pick up an object that fell to the ground and hand it back to the experimenter, and they open a cabinet so that an experimenter can put items in it (Warneken & Tomasello, 2006, 2007). While a focus on short action sequences suggests that these behaviors are helpful, a broader view on infants’ and toddlers’ activities suggests that they are not always very helpful (Carpendale, Kettner, & Audet, 2015). That is, although in household chores infants seem to engage in helpful behavior (e.g., by putting laundry into a box), they shortly thereafter go on with being unhelpful (e.g., putting the laundry out of the box).

These findings have led to an intense debate on the developmental origins and psychological mechanisms underlying young children’s helping behavior (e.g., Allen, 2015; Brownell, 2013; Carpendale & Hammond, 2016; Dahl, 2015; Eisenberg, VanSchyndel, & Spinrad, 2016; Michael & Szekely, 2017; Paulus, 2014; Warneken & Tomasello, 2009). One theoretical perspective proposes the presence of genuine other-oriented and moral concerns in infancy. More specifically, it has been proposed that infants are naturally altruistic (Warneken & Tomasello, 2009) and that their helping is based on an evaluation of others’ instrumental needs (Dunfield & Kuhlmeier, 2013). Relatedly, some researchers have discussed the presence of an innate moral core (Hamlin, 2013). According to this perspective, young children’s helping is motivated by a wish to alleviate others’ instrumental need.

Another theoretical view holds that the early forms of instrumental helping are indicative of a more general motivation to interact with others (Carpendale et al., 2015; Paulus, 2014). Carpendale and colleagues (2015) highlighted that we should be cautious when labeling these early instances of social coordination as ‘helping’ given that this label might suggest an interpretation that may not be warranted. In this line, Dahl and Paulus (2018) presented a theoretical framework on the ontogeny of human altruism in early childhood. According to this model, early forms of prosocial behavior are pre-altruistic as they are driven by an interest in social interaction or a preference for action fulfillment. The emergence of genuine empathic concern by the end of the second year marks the first instance of altruistic concern for others. By 3–4 years, children reason about normative obligations to help needy others. This indicates another level of altruistic concern that involves an evaluation of what is good and bad. Overall, this framework suggests developmental changes in the nature of young children’s helping behavior. One corollary of this view is that helping behavior becomes genuinely other-oriented and need-based in the course of further development.

Although knowledge on the developmental timeline of need-based helping would thus be very informative for the field, there is to date no direct empirical evidence. Previous empirical work is inconclusive with respect to this question. In their seminal study, Warneken and Tomasello (2006) showed that 18-month-old infants were more inclined to help an experimenter who wanted to achieve a goal compared to an experimenter who demonstrated that he was not interested in this particular activity or object. Likewise, Dunfield, Kuhlmeier, O’Connell, and Kelley (2011) relied on a control condition in which the experimenter deliberately put an object away and showed no interest in obtaining this object. These control conditions nicely demonstrate infants’ ability to distinguish intentional and accidental behavior, but they did not systematically disentangle the contribution of the factors neediness and interest. That is, infants' differential responding in these conditions could be due to their appreciation of others' interest, not an assessment of others' needs. Another line of research proposed that indirect physiological indicators such as pupil dilation are indicative for the existence of a prosocial arousal when confronted with others’ unfulfilled needs (for review see Hepach, 2017). Yet, others contended that a closer reading of this literature suggests that the findings could more readily be explained by a desire to interact with others or to comply with direct requests (Pletti, Scheel, & Paulus, 2017). Taken together, previous research did not directly assess whether infants’ early helping is informed by others’ needs or whether there is a developmental differences in how others’ needs affect young children’s helping. Knowledge about when in development children’s helping behavior is related to others’ material need would inform current theories on the ontogeny of helping and cooperative behavior.

### The current study

The current study was conducted to explore whether and when in development young children’s helping behavior is affected by others’ needs. One account would predict that even the earliest forms of helping indicate a concern for others’ needs and a motivation to alleviate others’ negative states. Another account proposed that the earliest forms of helping are not affected by others’ needs. Yet, in the course of early development, helping might become an activity subserved by altruistic concerns.

To this end, we assessed young children’s helping behavior when being confronted with a needy and a non-needy other. In order to not confound interest with neediness, both persons demonstrated an interest (e.g., looking and reaching for an object), while we manipulated the need for help. Given that young children’s helping depends on clear social signals and requests (e.g., Svetlova et al., 2010), both persons relied on such signals. We examined whether or not they preferentially helped the needy person. Given that by 18 months, children’s helping seems to be present across different tasks (Warneken & Tomasello, 2006), we decided to test 1.5-year-olds as youngest age groups. Based on theoretical considerations that genuine altruistic concerns emerge by 2–3 years (Dahl & Paulus, 2018), we decided to test 3-year-old children to reveal potential developmental differences.

## Methods

### Participants

We tested 101 participants; 50 1.5-year-old children (*M* = 19.4 months, range = 17–21 months; 23 boys) and 51 3.5-year-old children (*M* = 42.4 months, SD = 40–45; 25 boys). Based on about medium-sized effects in past research (e.g., Warneken & Tomasello, 2006) and a power of 95% to detect an interaction effect, an overall sample size of at least 54 participants was required. Two 1.5-year-old children had to be excluded to unexpected interruption of the study. Participants came from a larger European city. They were mostly white middle-class families living in an urban area. Participants were recruited by sending invitation letters to families with children in the appropriate age range. Informed consent for participation was given by the children’s caregivers. Parents received a monetary compensation for travel expenses and the children a gift for their participation.

### Stimuli

**Fig. 1:**
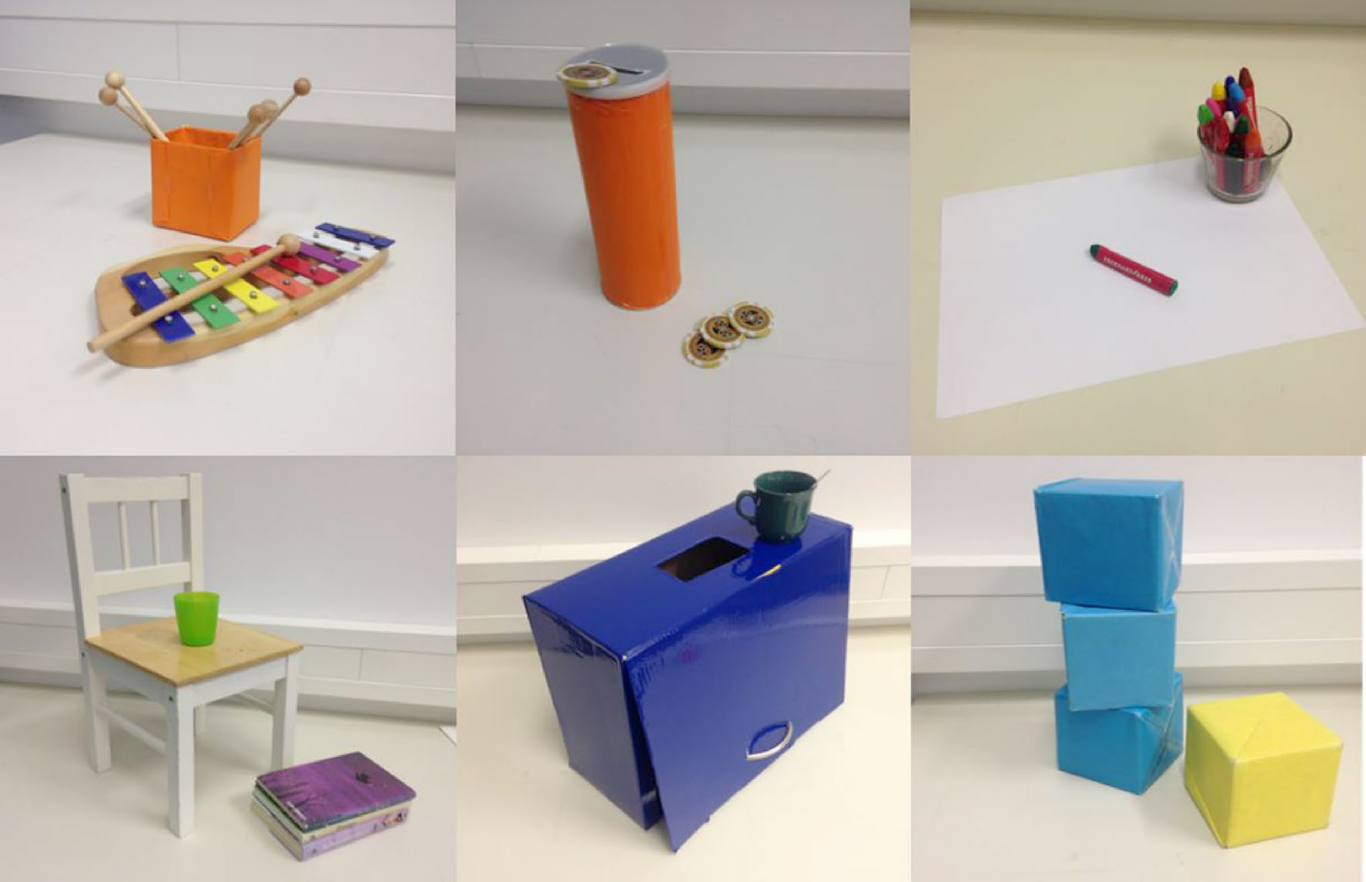
Snapshots of a selection of stimuli employed in the six different tasks

The experimental design relied on similar tasks (or slight variations thereof) that had been used in previous work on infant helping (e.g., Dunfield & Kuhlmeier, 2013; Paulus, Jung, O’Driscoll, & Moore, 2017; Warneken & Tomasello, 2006). Materials for the six tasks included the following objects: two xylophones, eight drumsticks, and a plastic box (xylophone task); two music boxes (height: 21.5 cm; diameter: 8 cm) containing a little bell and 20 toy-coins that could be inserted into the box (music box task); two sheets of paper, 13 crayons, and one glass (crayon task); two plastic cups, and two stacks of books (chair task); two flaps (44.5 cm × 36 cm × 19.5 cm) with a movable front lid and either a small (8.5 × 4.5 cm) or a large (12.5 × 6.5 cm) hole on their top, two cups, and two spoons (flap task); the same two flaps covered by white cloths, two yellow and six blue cubes (12 cm × 12 cm × 12 cm; cube task). For the first three tasks, two small tables (59 cm × 50 cm × 50 cm) were used, and for the first four tasks, two child chairs were employed. Figure 1 gives an overview on the some of the stimuli. In addition, a foam ball was used during familiarization.

### Experimental setup and procedure

Children were tested individually by three experimenters in a quiet room. Two cameras were used to record the sessions. The first experimenter (E1) welcomed the families, explained the study, collected parental consent, and familiarized children with the materials. The other two experimenters (E2, E3) played the role of the helpees. We decided to have a third experimenter taking over the introduction phase to avoid any bias on side of the children towards one of the experimenters who served as helpees.

*Familiarization phase* Children were first familiarized with all the materials involved in the study and their functionality by E1. For example, E1 demonstrated that inserting coins into a music box caused a bell sound. Moreover, she demonstrated that she was able to retrieve an object with her hand through the large opening on the top of one of the flaps, but had to use the front lid to get an object out of the other flap (as it had only a small opening on its top). Children were also allowed to interact with the items. Children’s parents were seated on a chair in the back of the room and were asked to not intervene during the entire familiarization and testing phase of the study. During this first familiarization phase, E2 and E3 remained silently in the back. Thereafter, E1 withdrew from the interaction and children were familiarized with E2 and E3 by jointly playing with the foam ball. We played close attention that the child rolled the ball to each experimenter several times and played with both experimenters to the same extent.

*Test phase* For the test phase, E1 left the room, and E2 and E3 ran the test trials. Since the xylophone task, the music box task, and the crayon task were administered by means of the table, these three tasks were always administered in a row (to avoid lengthy rebuilding of the setup after each trial). Thus, we balanced between participants whether they were first administered the three table-based tasks or the other three tasks. The order of the single tasks was also balanced between participants.

**Fig. 2:**
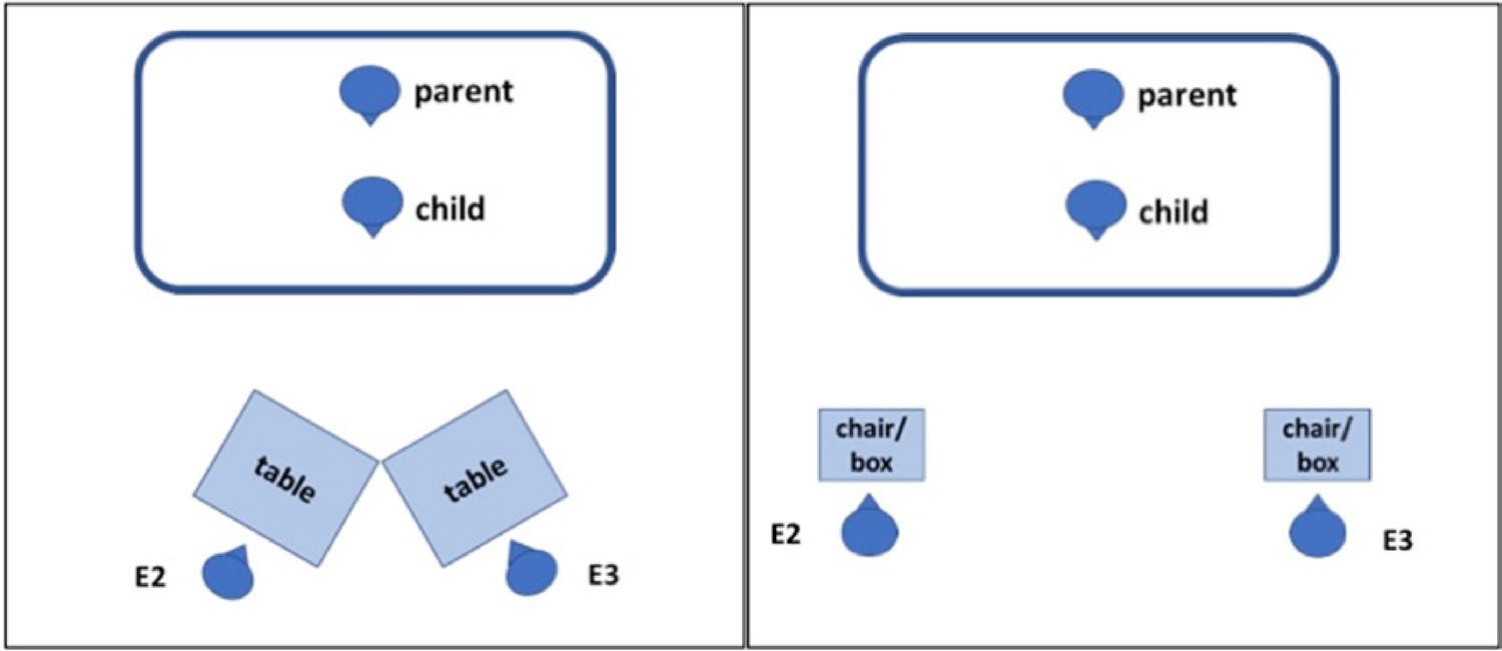
Schematic representation of the setups in the different trials

For the three table-based tasks, the two tables were situated in a 70° angle to each other (see also Fig. 2). E2 and E3 sat at a child chair behind one of the tables. In the xylophone task, each experimenter had a xylophone in front of her. One of the drumsticks was positioned at the location where the edges of both tables touched each other. Thus, it was at equal distance from E2 and E3. The other drumsticks were located at the other side of one of the two tables (i.e., very close to one of the experimenters, but not to the other). The experimenter with the additional drumsticks shall henceforth be labeled ‘non-needy’, whereas the other experimenter will be labeled ‘needy’. At the start of the trial, the experimenters talked to each other by saying: “Let us play with the xylophone.”—“Yes, let’s play with the xylophone.” The experimenters subsequently attempted to play with the xylophone by using their fingers. They continued: “It’s more fun with the drumsticks”—“Yes, it’s more fun with the drumsticks.”—“Where is a drumstick?”—“Yes, where is it?” Then, both experimenters looked at their xylophone (1 s), glanced over the table (1 s), looked at the drumstick located in the middle (1 s), said “Ah” (1 s), reached for the drumstick so that it accidentally fall from the table (1 s), and said “Oh” while looking at the drumstick (1 s). For the following 30 s E2 and E3 illustrated their wish for the drumstick by leaning over the table and reaching for the item with a longing expression (7 s), leaning back with a disappointed expression (7 s), and then repeating these two phases (14 s). Importantly, we kept expressions and utterances parallel for E2 and E3 to exclude the possibility that participants’ helping behavior could be driven by other features than the differences in need for help. If the child helped one of the experimenters, the experimenter took the object and continued the action. The experimenter did not thank the child or reward it otherwise. The procedure of the music box and crayon task were analogous to the xylophone task.

The chair task, flap task, and cube task followed the same procedure with the following differences. Instead of the two tables, two chairs (chair task) or two boxes (flap task, cube task) were located in a distance of 1.30 m to each other. In the chair task, E2 and E3 were carrying a stack of books (the needy experimenter held them with both hands, whereas the non-needy was able to carry them with one hand and had thus one hand completely free) and wanted to take a seat at their respective chair. Given that there was a plastic cup on each chair, E2 and E3 tried to remove it to be able to take a seat. In the flap task, E2 and E3 pretended to drink tea and accidentally dropped their spoons into their respective flap and looked for it with a sad face. In the cube task, E2 and E3 sat behind the box and built a tower out of the four cubes. They first used three blue cubes and placed the yellow cube on top of each tower when it accidentally fell to the ground. While the cube of the non-needy experimenter fell right next to the tower, the cube of the needy experimenter fell out of the experimenter’s reach. All tasks had the same 30-s phases as described above in which the experimenters expressed their wish for the respective object. We counterbalanced across trials whether E2 or E3 was the needy or non-needy person.

### Data coding and analysis

We coded for each trial whether or not the child showed helping behavior, and which of the two experimenters (needy, non-needy) the child helped. In the xylophone task, the music box task, and the crayon task, helping was defined as bringing the object to one of the two experimenters by either placing it into the hand or the table. In the chair task, helping was defined by removing the cup. In the flap task and cube task, helping was defined as returning the spoon or the cube, respectively, to the experimenter. Children’s completion of the experimenter’s intended action was also coded as helping (e.g., in the cube task, putting the cube back on the tower). In all cases children’s first behavior was coded. Trials that were interrupted or contained an experimenter error were excluded from further analysis. Overall, 34 trials (5.7%) had to be excluded. 29 participants were coded by a second rater. Interrater agreement for the six trials were *κ* = 0.93, *κ* = 1.00, *κ* = 0.88, *κ* = 0.91, *κ* = 1.00, and *κ* = 0.94, respectively.

For statistical analyses, we built average percentage scores on the number of trials in which participants helped the needy and the non-needy experimenter. Data were analyzed by a mixed-model analysis of variance (ANOVA) with age-group (1.5 years, 3.5 years) and gender as between-subjects factors and experimenter (needy, non-needy) as within-subjects factor. To further ensure that any tendency would not be masked by trials in which no helping occurred, we calculated the percentage of trials in which children helped the needy experimenter out of all trials in which helping occurred. That is, we disregarded all trials in which no helping occurred. Children who did not help in any of the trials were altogether removed from analysis. Given that helping the needy and non-needy experimenter logically sums up to 100% (after removal of trials in which no help occurred), this analysis was only focused on the percentage of trials in which children helped the needy experimenter. Thus, data were analyzed by an ANOVA with age-group (1.5 years, 3.5 years) and gender as between-subjects factors.

## Results

### Descriptives

Table 1A, B give the number of participants who helped the needy other, the non-needy other, or showed no helping in the respective tasks.

**Table 1:** Number of participants who helped the needy other, the non-needy other, or showed no helping behavior

	Xylophone	Music box	Crayon	Chair	Flap	Cube
(A) 1.5-year-old children						
Needy other	3	6	8	3	3	7
Non-needy	6	12	10	5	3	4
No helping	33	30	30	36	41	32
(B) 3.5-year-old children						
Needy other	16	16	20	10	12	14
Non-needy	12	15	9	6	10	10
No helping	20	20	20	33	29	20

### Confirmatory analyses

**Fig. 3:**
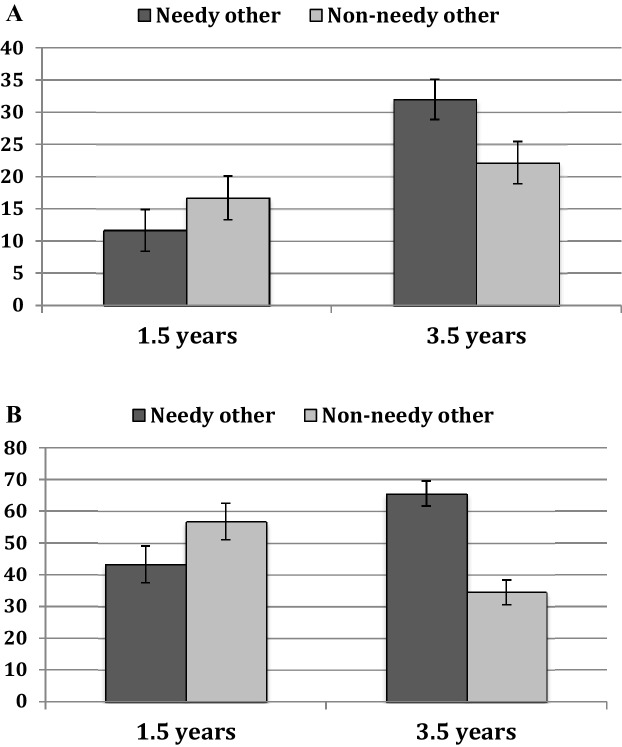
**a** Children’s helping rate across all valid trials. **b** Children’s helping rate if trials without a response were excluded. Error bars represent standard errors of the means

The ANOVA revealed a highly significant effect of age-group, *F*(1, 95) = 11.08, *p* = 0.001, *η*^*2*^ = 0.10, indicating that older children engaged in more helping behavior than younger children. This main effect was qualified by a significant interaction between age-group and recipient, *F*(1 ,95) = 8.84, *p* = 0.004, *η*^*2*^ = 0.09 (see Fig. 3a for means; all other *p*s > 0.34). Planned paired-samples *t *tests confirmed that the older children were more helpful towards the needy other than the non-needy other, *t*(50) = 2.69, *p* = 0.010, whereas there was no difference in the younger age group, *t*(47) = 1.47, *p* = 0.148. In addition, independent sample comparisons across age groups showed that the older children were more likely to help the needy other than the younger children, *t*(97) = 4.52, *p* < 0.001, whereas there was no difference for the non-needy other, *t*(97) = 1.18, *p* = 0.240. This shows a developmental increase in young children’s likelihood to help a needy other, but not a non-needy other.

The ANOVA on the participants that helped in at least one trial included 20 1.5-year-old children and 38 3.5-year-old children. It revealed only an affect of age-group, *F*(1, 54) = 11.08, *p* = 0.002, *η*^2^ = 0.17, indicating that older children were more likely to help the needy other than the younger children (see Fig. 3b). This result mirrors the finding of the previous analysis that included all trials. There was a non-significant tendency that girls (*M* = 60.18, SE = 4.7) were more likely to help the needy other than boys (*M* = 48.64. SE = 4.7), *F*(1, 54) = 2.96, *p* = 0.091, *η*^2^ = 0.05. The interaction was not significant, *F* < 1.

**Fig. 4:**
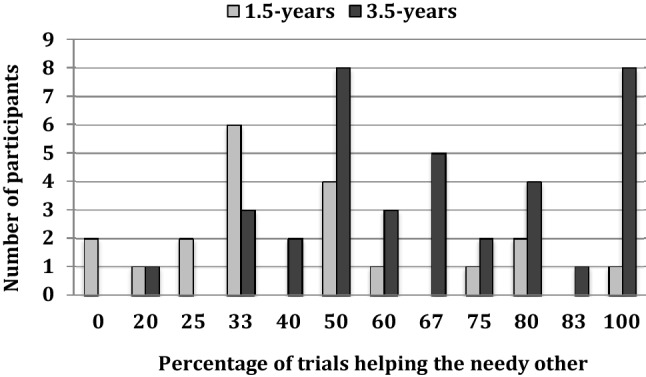
Number of participants (*y*-axis) who helped the needy other in a particular percentage of trials. Note that the different percentage values are a consequence of different trial numbers being included for different participants

To ensure that younger children’s non-selective helping behavior was not consequence of one subgroup of 1.5-year-olds showing consistent helping towards the needy other, whereas the other subgroup showing consistent helping towards the non-needy other, we plotted the distribution of the trials (in percentages; that is, out of the trials in which help was provided) in which participants helped the needy other. Figure 4 shows that there was no such bimodal distribution in young children.

### Further coding

To ensure that the differences in children’s helping were not driven by young children not attending to either of the two recipients or even both recipients, we coded whether participants attended either of them. More concretely, we coded whether (1) they attended the respective recipient, whether they (2) did not attend the respective recipient, or whether (3) their looking behavior was not codable. The analyses showed ceiling effects for the 1.5-year-old children for both the needy other (attending: 98.6%; non attending: 1%; not codable: 0.4%) and the non-needy other (attending: 99.3%; non attending: 0%; not codable: 0.7%). Likewise, there were ceiling effects for the 3.5-year-old children for both the needy other (attending: 98.7%; non-attending: 0.7%; not codable: 0.7%) and the non-needy other (attending: 99.3%; non-attending: 0.3%; not codable: 0.3%). Thus, children of both age groups attended to both recipients in the vast majority of trials.

## Discussion

This study examined whether or not, and from which age on, young children consider other’s action-related needs in a cooperative helping task. More concretely, we investigated whether 1.5- and 3.5-year-old children are more likely to help a needy other than a non-needy other. Our results show a clear developmental effect with only the 3.5-year-old children, but not the 1.5-year-old children selectively helping a needy other. This finding is in line with theoretical proposals that early helping is not motivated by a consideration of others’ needs, but becomes a genuinely need-oriented behavior in the course of early childhood.

It is well known that young children start to engage with others in coordinated activities in their first year of life. These activities include simple social games such as peekaboo (Bruner & Sherwood, 1976), but also social routines such as postural adaptations when being picked up (Reddy et al., 2013) and self-care tasks (Hammond et al., 2017). In the course of the first year, coordinated activities proceed from dyadic to triadic interactions that involve joint attention to objects (Moore, 2006). Around the same age, children start to engage in simple instrumental helping behaviors (Warneken & Tomasello, 2007) that can also be regarded as instances of triadic interactions between two persons and an object. Yet, their coordinated activities largely depend on adults’ scaffolding (Brownell, 2011), are enhanced by adult encouragement (Dahl, 2015), and are embedded in established social routines (Barragan & Dweck, 2014). Further in development, children become increasingly able to engage in efficient social coordination without further scaffolding (Brownell, Ramani, & Zerwas, 2006; Meyer, Bekkering, Paulus, & Hunnius, 2010; Milward, Kita, & Apperly, 2014; Satta, Ferrari-Toniolo, Visco-Comandini, Caminiti, & Battaglia-Mayer, 2017). Our study adds to this line of research on the development of social coordination the finding that instrumental helping undergoes developmental changes in early childhood. The finding that it is by 3 years that children start to appreciate others’ needs relates well to other studies reporting difficulties in efficient joint action performance in young children (e.g., Milward et al., 2014; Paulus, 2016; Satta et al., 2017).

It should be noted that our pattern of results remained the same even when excluding the children who did not engage in helping behavior at all. Moreover, a coding of children’s viewing behavior indicated that they looked at each helpee rendering it unlikely that infants’ behavior could be due to ignoring one of the helpees. In addition, although our study was not designed to compare the different trials, an inspection of the descriptives indicates a uniform pattern across the different trial types for each age group. Finally, given our large sample it is unlikely that the finding with the younger children was due to insufficient power.

### From early social interaction to other-oriented behavior

The current study suggests that 1.5-year-old children’s helping is not affected by others’ need. These findings relates well to other findings on limitations in young children’s helping. It has been shown that 24-month-old, but not 14-month-old infants use information about the goal object of an actor’s previous reaching behavior to hand him the respective object in a subsequent helping task (Hobbs & Spelke, 2015). Notably, 14-month-old infants only hand over an object an experimenter is currently reaching for. In line with this finding, Paulus and colleagues (2015) reported that goal encoding at 7 months did not predict instrumental helping at 18 months. Furthermore, most studies on infant helping rely on warm-up phases in which infants are familiarized with the social routine of handing over objects to an experimenter before being assessed in test trials. It has been shown that without such a previous social interaction phase, infants’ helping is drastically reduced (Barragan & Dweck, 2014). Overall, these results indicate that infants’ helping behavior is based on responding to signals in social interactions. The pattern of findings is in line with views that infants’ helping behavior emerges out of established action routines (Dahl, 2015; Hammond, 2014), and could be driven by a social motivation to interact with others (Carpendale et al., 2015; Paulus, 2014).

These early forms of social coordination with others become transformed into genuinely other-oriented helping behavior in the course of the next years. A few findings indeed point to developmental changes in young children’s helping behavior in the course of toddlerhood. For example, Dahl and colleagues (2017) showed that for 13- to 15-month-old infants explicit scaffolding (e.g., praise) led to increased helping, whereas 16- to 18-month-olds were not affected by scaffolding. Interestingly, by 20 months, rewards could even reduce children’s propensity to help (Warneken & Tomasello, 2008). These findings point to the role of socialization in the early emergence of helping (for reviews see Brownell et al., 2016; Dahl, 2018) and suggest that by the end of the second year, helping behavior is an established routine. When they are prevented from helping themselves, 2.5-year-old toddlers (but not 1.5- to 2-year-olds) systematically involve their own caregivers to help a needy other (Paulus et al., 2017; see also Karasevich, Kuhlmeier, Beier, & Dunfield, 2018), indicating a motivation to see others being helped. Similarly, by 2 years toddlers show signs of proactive helping, that is, help spontaneously by putting an object back on a table without being requested to do so (Warneken, 2013). By 3.5 years, children do not hand over a requested object when this item is dysfunctional and therefore unlikely to be actually helpful (Martin & Olson, 2013). Rather, they choose a different object that is a better alternative for helping her to achieve her goal. Our results add to these findings by demonstrating that by 3.5 years, children’s instrumental helping is affected by others’ needs.

Overall, these results could suggest that helping behavior becomes an other-directed and need-oriented behavior in the course of early development. This interpretation is in line with recent theoretical claims that human altruism emerges gradually in early childhood (Dahl & Paulus, 2018; see also Bar-Tal, 1982). Gaining a fuller understanding of the emergence of other-oriented helping requires a closer examination of the developmental pathways connecting the first instances of helping with later forms of other-oriented helping behaviors.

From a general theoretical point of view, acts of instrumental helping appear to be another prime example of the idea that the same kind of behavior has different meanings and different underlying mechanisms at different points in development (Fischer & Bidell, 2006). One possibility is that these early forms of helping behavior gain their specific prosocial meaning and need-directedness by experiencing their meaning in social interactions, i.e., by learning of how others react to their helping behavior. That is, action and action experiences would be the main factors driving cognitive development (Allen & Bickhard, 2013). If this were true, it would provide a mechanism of how early helping could become an instance of reflected activity (e.g., considering the consequences of one’s own actions) in the course of the first years of life (Hay & Cook, 2007).

### Limitations and open questions

Although the current study suggests that by 3 years children’s helping is informed by others action-related needs, it leaves open the precise nature underlying younger children’s helping. We offer several (not mutually exclusive) considerations: First, a general motivation to jointly interact with others could support their engagement in the social context without considering the consequences of this action. Indeed, young children like to engage with others in joint activities and experience pleasure by social exchange (Moore, 2006). Second, in some of the tasks, the actors demonstrated a desire for an object. It seems thus plausible to assume that younger children’s helping is triggered or supported by clear signs of desires for objects. Indeed, some studies relied on clear signs such as extended hand to demonstrate early helping and sharing in infants (e.g., Dunfield & Kuhlmeier, 2013). Moreover, it has been shown that young children often need clear and explicit communicative cues to engage in helping (Svetlova et al., 2010) and sharing (Brownell, Svetlova, & Nichols, 2009). Finally: A closer look at the different tasks indicates that in the three table-based tasks (xylophone task, music box task, crayon task) young children showed a descriptive pattern of rather helping the non-needy other than the needy other. Notably, in these tasks need was manipulated by having or not having several tokens of the required items. For example, in the crayon task the non-needy other had a couple of crayons on her table. This pattern of preferentially handing over items to persons who already possess enough resources has also been noted in studies on young children’s sharing (Rizzo & Killen, 2016). It might relate to a tendency to collect items or put them together. Indeed, some helping behaviors in household chores involve cleaning up routines (Carpendale et al., 2015; Dahl, 2015; Rheingold, 1982). Moreover, it could be related to an affective preference for a lucky and wealthy other (Li, Spitzer, & Olson, 2014). Yet, given that this pattern was not evident in the other tasks, it cannot explain the overall pattern of our finding.

The current study has a number of limitations and leaves open some questions. First, although a closer analysis of children’s looking behavior revealed that children of both age groups attended the recipients at the vast majority of trials, rendering it unlikely that participants did not visually attend the scenery, we cannot say for sure how exactly participants processed the visual information. Post hoc speculations that although children attended to the different need states, they did not register it or decided to not act based on it, cannot be excluded and require future research. Second, younger children’s overall helping rate was rather low. Yet, also other studies reported that young children often do not engage in helping (Waugh & Brownell, 2017). Descriptively, it seems that they were more likely to help in the three table-based out-of-reach tasks. This corresponds to other studies that also reported higher rates of helping in simple out-of-reach tasks (Warneken & Tomasello, 2006, 2007). Finally, in order to defend an altruistic interpretation of young children’s helping, one could argue that the helpee’s reaching behavior might have triggered infant helping and distracted from the differences in material need. However, this alternative explanation is problematic for two reasons. First, from a theoretical point of view, this argumentation could suggest that infants’ helping is mainly triggered by their compliance with social requests rather than differences in the helpees’ need. Yet, this would in itself not be in line with an account that assumes that early helping is directly indicative of altruistic acts and based on an assessment of others’ need. Second, not all of our tasks involved reaching actions. For example, in the chair task neither of the adults reached. Nevertheless, the result pattern was the same. This finding renders it unlikely that infants’ equal treatment of both helpees is merely due to being triggered by their reaching behavior. Of course, it is possible to come up with additional post hoc ideas of why infants were equally likely to help the needy and non-needy other, for example, that infants perceived others’ need, but were (in contrast to toddlers) not able to compare it. Given that no current theory represents such a view, from a scientific point of view (Popper, 2002), these ideas would have the epistemological status of speculations that would require independent empirical substantiation in future studies; and would likely require a modification of current theories on early social development to become theory-guided testable predictions.

One general concern relates to the question of how to describe young children’s behavior in helping contexts in a neutral way. Carpendale and colleagues (2015) highlighted that “labeling toddlers’ behavior as helping is already jumping to a conclusion about its nature. Caution is required to avoid describing infant and toddler behavior in terms of adult understanding” (p. 359). There is certainly a danger in over-interpreting young children’s behavior merely by choosing descriptions or labels that bear specific meanings. It would indeed be safer to label these phenomena as instances of ‘social coordination’. Yet, if we want to stick to the label ‘helping’, we need to be careful to not attach implications merely by using this description.

In sum, the current study contributes to our knowledge on the development of cooperative behavior in young children. It demonstrates that 3-year-old, but not 1.5-year-old children’s helping is affected by others’ need. Thus, young children’s behavior becomes increasingly sensitive towards the environmental factors that are important to consider in successful social interactions.
